# Expression of *RcHSP70*, heat shock protein 70 gene from Chinese rose, enhances host resistance to abiotic stresses

**DOI:** 10.1038/s41598-020-58745-6

**Published:** 2020-02-12

**Authors:** Changhua Jiang, Yuke Bi, Ruyao Zhang, Shucheng Feng

**Affiliations:** Shanghai Botanical Garden, Shanghai, China

**Keywords:** Molecular biology, Abiotic

## Abstract

There exist differences in the heat tolerance of Chinese rose varieties, and high temperature in summer can lead to failure of blooming in non-heat-tolerant Chinese rose varieties. We cloned a heat shock protein 70 gene (designated *RcHSP70*) from heat-tolerant varieties of Chinese rose (*Rosa hybrida* L.) to elucidate the molecular mechanism of heat tolerance and improve the quality of Chinese rose. Degenerate primers were designed for *RcHSP70* according to the 5′- and 3′-end sequences of *HSP70* genes in apple and tea. *RcHSP70* was cloned from heat-tolerant Chinese rose varieties after heat shock. The heat shock-induced expression patterns of *RcHSP70* in different Chinese rose varieties were analyzed by RT-PCR. Following heat shock (38 °C/3 h), *RcHSP70* was highly expressed in the heat-tolerant varieties but not in the non-heat-tolerant varieties, indicating a close relationship between *RcHSP70* and heat resistance in Chinese rose. To verify the function of *RcHSP70*, we constructed a prokaryotic expression recombinant vector for this gene and transformed it into *Escherichia coli* BL21. The tolerance of recombinant strains to abiotic stresses, including high temperature, low temperature, high salt, heavy metals, high pH, and oxidation, was evaluated. Additionally, *RcHSP70* was transformed into tobacco plants. Because of the overexpression of this gene, transgenic tobacco plants improved their tolerance to high temperature and cold. In addition, transgenic tobacco showed better photosynthetic performance, relative electrical conductivity and proline content than wild tobacco after heat stress and cold stress. Our findings indicate that *RcHSP70* is involved in the resistance of Chinese rose to abiotic stresses.

## Introduction

High temperature in summer often severely restricts the growth and development of Chinese rose (*Rosa hybrida* L.). So Chinese rose in the high temperature season on the general existence of the following problems: (1) The high temperature in summer causes the flower buds of Chinese rose to stop differentiation and transform into vegetative growth, which results in the heat-resistant varieties not flowering and poor growth in summer. (2) Due to high temperature and humidity in summer, diseases and insect pests are aggravated, such as black spot disease, powdery mildew is particularly common, seriously affecting the growth and development of Chinese rose^1^. Therefore, the study of heat tolerance in Chinese rose is significant.

Heat shock proteins (HSPs) are a class of stress proteins that undergo inducible synthesis in organisms under environmental stress, such as high temperature, salt, drought, starvation, and heavy metal ions. High temperature is the main factor that induces HSP synthesis^2^. In addition to heat stress, other environmental stress factors, such as drought, low temperature, and heavy metal ions, can upregulate HSPs in plants^3^.

Molecular chaperones are proteins that are associated with the folding of a nascent peptide chain, as well as the assembly and transport of proteins. Many *in vitro* and *in vivo* studies have shown that HSPs act as molecular chaperones in cells^3,4^. Heat shock protein 70 is one of the main members of heat shock protein family. The heat shock protein 70 family mainly consists of four members, which are inducible heat shock protein 70, constitutive heat shock protein 70, glucose regulatory protein 78 and GRP75. Among them, induced heat shock protein 70 was not expressed or low in normal conditions, but its expression increased sharply after treatment with temperature and other stress factors. Heat shock protein 70 plays an important role in biological resistance to temperature stress. overexpression of HSP70 improves heat tolerance in plants^5^. Heat shock protein 70 has been studied more in animals, but less in plants.

Jiang *et al*. treated Chinese rose plants of the heat-tolerant variety ‘Schloss mannieim’ and the non-heat-tolerant variety ‘Kordes Perfecta’ at normal temperature or by heat shock at 38 °C for 3 h^6,7^. They extracted the soluble total proteins from young leaves and obtained protein spots that were expressed specifically in heat-shocked ‘Schloss mannieim’ plants by two-dimensional electrophoresis. One spot was identified to be a small-molecule HSP, based on peptide analysis by mass spectrometry^8^. These findings prompted us to further investigate the heat resistance of Chinese rose. We obtained the full-length cDNA sequence of the open reading frame (ORF) of Chinese rose HSP70 (designated RcHSP70) by homologous cloning. RcHSP70 comprised 1956 bp, including 651 amino acids with a deductive molecular weight of 71.1. We analyzed the heat-induced expression patterns of RcHSP70 and its responses to multiple abiotic stresses in different Chinese rose varieties.

## Materials and Methods

### Materials

In early May 2015, we selected the heat-tolerant Chinese rose varieties ‘Schloss mannieim’ (SM), ‘Radio’ (RA), ‘Las vegas’ (LA), and ‘Girl’ (GI) and the non-heat-tolerant varieties ‘Kordes Perfecta’ (KP) and ‘Pfalzer gold’ (GO) from the Chinese rose garden of Shanghai Botanical Garden (according to the phenology records of Shanghai Botanical Garden; data not shown). All plant materials were potted seedlings, Grafted for two years. All strains used were bought from Suo Lai Bao biology Lo., Co. (Shanghai, China) and maintained in the Research Center of Shanghai Botanical Garden. Taq plus was purchased from Sangon (Shanghai, China). A PrimeScript™ RT Reagent Kit, restriction enzymes, and T4 DNA ligase were purchased from Takara (Qindao City, Shandong Province, China). A gel extraction kit was purchased from Shennengbocai Biotechnology Lo., Co. (Shanghai, China). Marker DL2000 was purchased from Tianwei Biotechnology Lo., Co. (Shanghai, China). Other reagents were analytical grade reagents made in China or overseas.

### Analysis of high-temperature stress-induced *RcHSP70* expression

All heat-tolerant and non-heat-tolerant Chinese rose varieties were treated at 38 °C for 3 h before young leaf samples were collected. Total RNA was extracted and purified using the Plant RNAout Kit (Tianze Genetic Engineering Co., Ltd.). First-strand cDNA was synthesized by using PrimeScript™ RT Reagent Kit (TaKaRa)^9^. The forward and reverse degenerate primers for *RcHSP70* were designed according to the 5′-end and 3′-end sequences of the ORF of apple *HSP70* (AF161180) and tea *HSP70* (EU714122): 5′-ATGKCVGGAAAGGGAGAGG-3′ and 5′-TTARTCAACTTCYTCRATCT-3′. PCR was performed using the synthesized first-strand cDNA as template. The PCR program comprised: pre-denaturation at 94 °C for 4 min; followed by 30 cycles of denaturation at 94 °C for 40 s, annealing at 55 °C for 40 s, and extension at 72 °C for 2 min; and a final extension at 72 °C for 10 min. The material at normal temperature (25 °C) was used as a control.

### Prokaryotic expression of *RcHSP70*

#### Construction of prokaryotic expression vector

Primers with adapters were designed according to the 5′-end and 3′-end sequences of the ORF of *RcHSP70*. An *Eco*R V restriction site was introduced into the forward primer, and a *Sal* I restriction site was introduced into the reverse primer. PCR was carried out with cDNA as template using the same PCR program as in subsection 1.2. The PCR product and pET32a were double-digested with *EcoR* V and *Sal* I. The digested products were recovered and ligated using T4 ligase to generate a recombinant vector, pET32a-RcHSP70. The ligation product was transformed into *E. coli* DH5α, and the sequencing data revealed that the *RcHSP70* cDNA sequence was inserted correctly without any frameshift. The pET32a-RcHSP70 recombinant plasmid and pET32a (empty vector, EV) were extracted and transformed into *E. coli* BL21. Positive recombinant strains were stored at −80 °C until use.

#### Analysis of temperature resistance in the recombinant BL21strain

A 1-mL sample of bacterial fluid, induced with isopropyl β-D-thiogalactoside (IPTG) for 2 h, with an optical density of 1.0 at 600 nm (OD_600_), was centrifuged at 4500 rev. min^−1^ for 5 min. The cell pellet was resuspended in an equal volume of sterile water (1 mL of the suspension contained 10^9^ cells when the OD_600_ reached 1.0). A 100-µL sample of the resuspension was diluted to 1 mL. After being mixed, 100 µL of the mixture was taken and diluted to 1 mL. This step was repeated six times (to 10^3^ cells/mL). Subsequently, 100 µL of the diluted culture solution was spread onto Luria-Bertani (LB) agar plates (containing 100 mg L^−1^ ampicillin). The plates were incubated in the dark at 4 °C for 0, 2, 4, 6, 8, and 12 d and then kept at 37 °C overnight. The number of colonies on each plate was counted; this experiment comprised three replicates.

A separate bacterial fluid, induced with IPTG for 2 h with an OD_600_ of 1.0, was transferred to 55 °C, further induced, and cultured under high-temperature stress^11^. One-mL samples were taken at 0.5, 1, 2, and 3 h. After centrifugation, the cell pellet was resuspended with sterile water to an OD_600_ of 1.0. The samples were diluted sequentially, spread onto agar plates, and incubated at 37 °C overnight. The number of colonies on each plate was counted; the experiment comprised three replicates.

#### Analysis of resistance in the recombinant BL21 strain to other stresses

The recombinant BL21 strains containing pET32a-RcHSP70 (H1 and H2, parallel samples) and pET32a (EV) were cultured and induced with IPTG for 2 h until the OD_600_ reached 1.0. The wild-type BL21 (WT) strain was cultured and induced in LB broth as a parallel control. The culture was centrifuged (4500 rev min^−1^, 5 min), and the cell pellet was resuspended in sterile water (OD_600_ = 1.0). An inoculating loop was used to spread the culture in a Z-shaped pattern from inside to outside on LB plates with different levels of resistance. The plates were incubated at 37 °C overnight to monitor colony growth. The following reagents were added to solid LB medium to prepare LB plates with different levels resistance: (1) 100, 200, 300, and 400 mmol L^−1^ LiCl; (2) 400, 500, 550, and 600 mmol L^−1^ NaCl; (3) 10, 15, 20, and 25 mmol L^−1^ Na_2_CO_3_; (4) 300, 350, 400, and 450 mmol L^−1^ CdCl_2_, and (5) 200, 300, 400, and 500 μmol L^−1^ H_2_O_2_.

### Analysis of resistance of transgenic tobacco to temperature stress

#### Construction of plant expression vector

Total RNA was extracted from young leaf samples of SM after heat shock treatment (38 °C/3 h) using the Plant RNAout Kit (Tianze Gene Engineering Co., Ltd.). First-strand cDNA was then synthesized using the PrimeScript™ RT Reagent Kit (TaKaRa)^9^. The 5′-end forward and 3′-end reverse primers, respectively, were designed according to the 5′-end and 3′-end sequences of the ORF of *RcHSP70*. The 5′-end forward primer was 5′- CGGAGCTCatggccggaaagggagag-3′ (with a *Sac* I restriction site), and the 3′-end reverse primer was 5′-CGTCTAGATTAGTCAACTTCTTCTAT-3′ (with an *Xba* I restriction site). PCR was performed with cDNA as template. The PCR products were recovered and ligated to the PHB vector to construct a plant expression vector, PHB-RcHSP70. The sequencing results showed that the sequence of the inserted *RcHSP70* fragment was correct, without any frameshift.

#### Transformation of *RcHSP70* into tobacco and screen for positive lines

The recombinant plasmid PHB-RcHSP70 was transformed into *Agrobacterium tumefaciens* strain GV3101 and then transformed into tobacco leaves by *Agrobacterium*-mediated infection (leaf disk method)^1^. Positive plants were screened using hygromycin (15 mg/L) to obtain the T_0_ generation of transgenic tobacco plants (L1). To determine whether the transgenic tobacco plants (L1) constitutively overexpressed *RcHSP70*, RT-PCR was conducted per Molecular Cloning: A Laboratory Manual (Third Edition)^1^, with actin as an internal reference. Western blot was performed to determine whether the transgenic tobacco plants (L1) constitutively overexpressed RcHSP70. The primary antibody was anti-RcHSP70, prepared by Shanghai Sangon Biological Co., Ltd. The secondary antibody was sheep anti-rabbit IgG that was labeled with horseradish peroxidase. Wild-type tobacco was used as a blank control.

#### Analysis of resistance to high temperature and cold stress in transgenic tobacco plants

Transgenic tobacco plants of the five-leaf stage were selected for heat shock treatment (46 °C/2d), and we determined whether the resistance to high temperature stress improved. In addition, the plants were subjected to cold stress (−5 °C/2d) to determine whether the resistance to cold stress improved. Wild-type tobacco was used as a blank control.

### Analysis of physiological indexes of heat resistance in transgenic tobacco plants

Transgenic tobacco plants of the five-leaf stage were selected for treatment ①: 46 °C/5 h and ②: −5/5 h.

#### Analysis of changes in relative photosynthetic indexes

Leaf net photosynthetic rate (*P*_*n*_), intercellular carbon dioxide concentration (*C*_*i*_) and stomatal conductance (*G*_*s*_) of transgenic tobacco plants treated as ① and ② were measured by photosynthetic instrument Li-6400 (LI-COR, USA), under the illumination intensity 1000 Lux outdoor. Wild-type tobacco was used as a blank control.

#### Measurement of relative electrical conductivity

Electrical conductivity was measured by following the method in the “Experimental Guide for Plant Physiology”. Briefly, one leaf sample was selected from the same position on each plant treated as ① and ②. Three discs were punched from the selected leaf using a puncher (Ф = 0.8 cm) and placed in 7 mL of deionized water. The samples were vacuumed until the leaf discs were completely immersed. The samples were then allowed to stand at room temperature (25 °C) for 5 h before measuring electrical conductivity (T1). To measure the absolute electrical conductivity, the samples were incubated in a boiling water bath for 5 min and then cooled to room temperature. The electrical conductivity (T2) was then measured. Then the relative conductivity is calculated by T1/T2 × 100%.

#### Measurement of proline content

The proline content was measured according to the method in the “Experimental Guide for Plant Physiology” with slight medications. In brief, leaf samples were selected from the same position on each plant after treatments ① and ②. The samples were cut into pieces and mixed. A 0.2-g portion was weighed out and 2.5 mL of 3% (w/v) sulfosalicylic acid was added. The sample was incubated in a boiling water bath for 10 min. After cooling, 1 mL of the supernatant was taken, and 1 mL of acid ninhydrin and 1 mL of glacial acetic acid were added. After mixing, the sample was incubated in a boiling water bath for 30 min. After cooling, 4 mL of toluene was added to produce a red substance and the solution was shaken well. The optical density of the solution was measured at a wavelength of 520 nm.

All above physiological indicators in the research were measured three times. The average value and standard deviation were calculated by using Excel 2007 software.

## Results

### Temperature-induced expression patterns of *RcHSP70*

**Figure 1:**
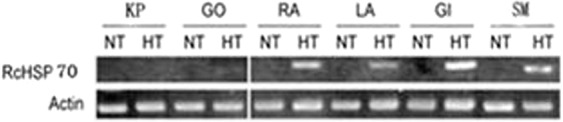
RT-PCR assay of heat shock-induced expression of RcHSP70 in rose varieties. NT- normal temperature(25 °C); HT- high temperature(38 °C/3 h); KP- Kordes’ Perfecta; GO- Pfalzer gold; RA- Radio; LA- Las Vegas; GI- Girl; SM-Schloss mannieim.

The RT-PCR results revealed that *RcHSP70* was not expressed in any variety at normal temperature (25 °C). Under heat shock conditions (38 °C/3 h), *RcHSP70* was highly expressed in the heat-tolerant varieties RA, LA, GI, and SM but not in the non-heat-tolerant varieties KP and GO (Fig. 1). These results indicate that this gene undergoes heat-induced expression in Chinese rose varieties.

### Resistance of recombinant strains to high and low temperature

**Figure 2:**
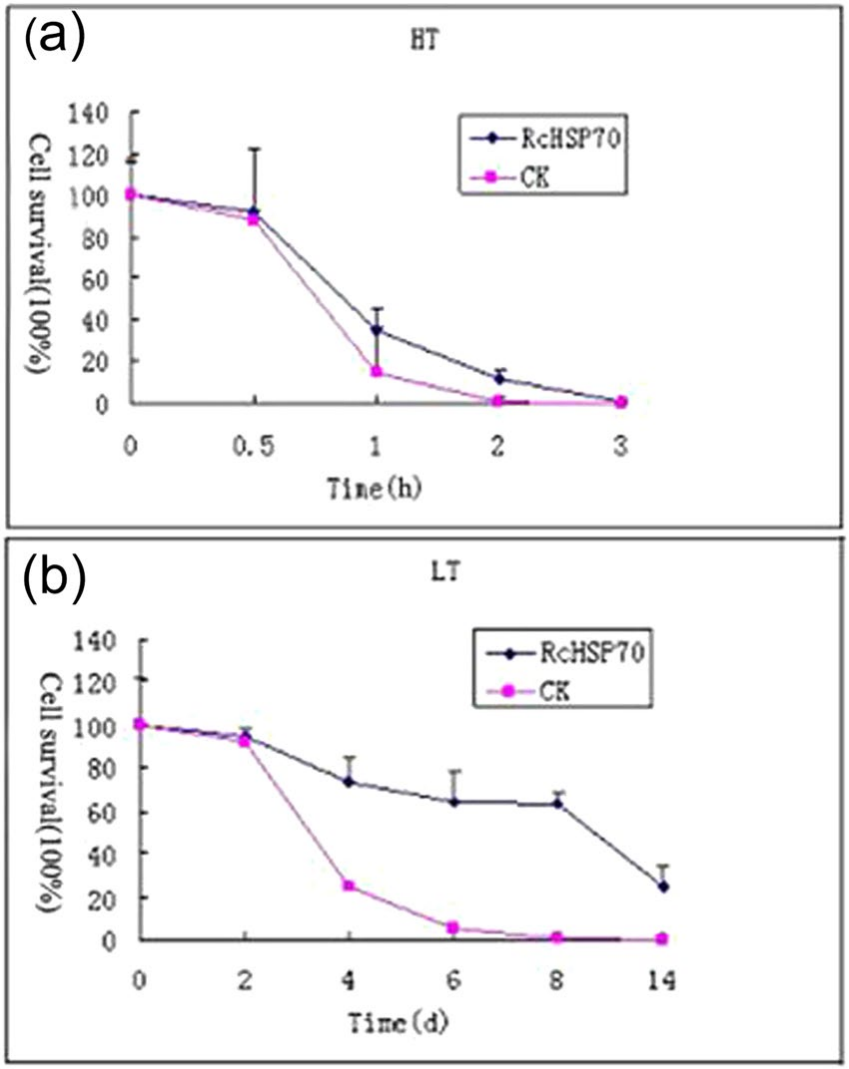
Resistance of tecombinant strains to high and low temperature stresses with the empty-vector strain as CK. (**a**) Viability of *E. coli* at 55 °C (**b**) Viability of E. coli at 4 °C.

After 0.5, 1, 2, and 3 h of high-temperature stress at 55 °C, the cultures were incubated at 37 °C overnight. High-temperature stress at 55 °C had a serious impact on the survival rate of the transgenic and EV strains. With increasing time of heat shock, the growth of transgenic and EV strains decreased sharply, but the rate of decline in the colony number of the EV strain was markedly higher than that of the transgenic strain. After 1 h of heat shock, the colony number of the EV strain dropped to 15.24%, whereas that of the transgenic strain was 35.12%. When the time of heat shock increased to 3 h, all colonies of the EV strain died, whereas 1.31% of the transgenic strain still survived. These experimental results indicate that the transformation of *RcHSP70* improves the tolerance of recombinant strains to high-temperature stress (Fig. 2a).

After 2, 4, 6, 8, and 14 d of low-temperature stress at 4 °C, the cultures were incubated at 37 °C overnight, and the number of colonies was counted. With increasing time of low-temperature stress, the colony numbers of the transgenic and EV strains fell constantly. However, the decrease in the colony number of the pET32a-RcHSP70 strain was slower compared with the rapid decease in the EV strain. After 14 d, the colony number of the EV strain dropped to 0% of the control, whereas that of the pET32a-RcHSP70 strain was maintained at 24.62%. These results indicate that the transformation of *RcHSP70* markedly improves the tolerance of *E. coli* to low temperature (Fig. 2b).

### Resistance of recombinant strains to other abiotic stresses

**Figure 3:**
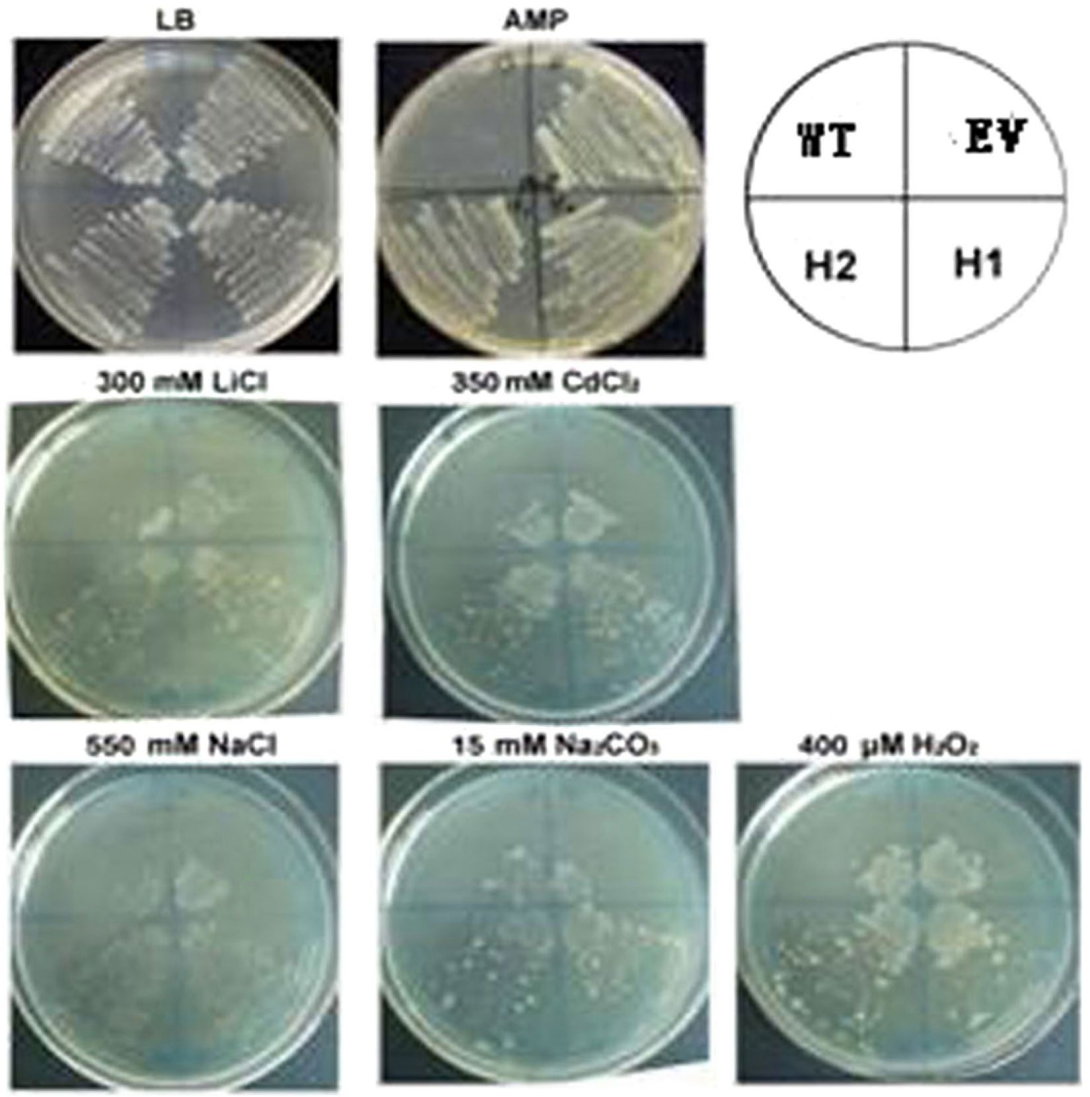
Viability assay of *E. coli* under multiple stresses. WT: Wild-type strain, EV: empty-vector strain, H1 and H2: RcHSP70 transgenic strain.

As shown in Fig. 3, a low concentration of LiCl did not significantly inhibit the growth of WT, EV, and *RcHSP70* strains (H1 and H2). When the concentration of LiCl reached 300 mmol·L^−1^, the growth of H1 and H2 was ~50%, whereas that of WT and EV was lower than 10%. With 350 mmol·L^−1^ CdCl_2_, the growth of WT and EV was severely inhibited to ~10%; the growth of H1 and H2 was inhibited to a lesser extent (~60%), with colonies nearly growing to the edge of the plate. These experiments show that because of overexpression of *RcHSP70*, the recombinant strains improve their resistance to heavy metal stress.

When the NaCl concentration rose to 550 mmol·L^−1^, many colonies of H1 and H2 continued to grow (~70%); in contrast, the growth of WT and EV was severely inhibited, indicating that the recombinant strains gained tolerance to high salt. At Na_2_CO_3_ concentration of 15 mmol·L^−1^, H1 and H2 colonies were able to grow to the edge of the plate (~40% of control); however, the growth of WT and EV was markedly inhibited (<10%). These results indicate that the recombinant strains increased their tolerance to high-pH stress.

The growth of all four strains was impeded by 400 μ mol·L^−1^ H_2_O_2_: few WT and EV colonies appeared at the starting position (~15%), and H1 and H2 colonies were able to reach the edge of the plate (~45%). These results indicate that the recombinant strain that undergoes inducible expression of *RcHSP70* has greater resistance to oxidative stress. All above percentages for colonies growing to the edge of the plate were supposed to 100%.

These experimental findings reveal that because of inducible expression of the fusion protein RcHSP70 in the recombinant strain (pET32a-RcHSP70), the accumulation of RcHSP70 improves the resistance of *E. coli* BL21 to abiotic stresses, including heavy metals, high salt, high pH, and oxidation. These findings suggest that RcHSP70 can respond to multiple abiotic stresses.

### Molecular identification of transgenic tobacco and analysis of resistance to temperature stress

#### RcHSP70 expression assay of transgenic tobacco

**Figure 4:**
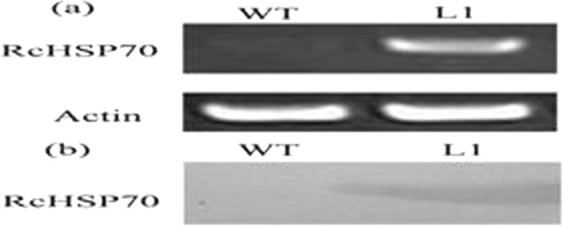
RcHSP70 expression level in wild-type(WT) and transgenic tobacco(L1) (**a**) RT-PCR test; (**b**) Western Blot.

By semi-quantitative RT-PCR, *RcHSP70* was constitutively overexpressed in transgenic tobacco line L1 (Fig. 4a).

Western blotting revealed constitutive overexpression of RcHSP70 in transgenic tobacco line L1 (Fig. 4b).

#### Resistance to temperature stress in transgenic tobacco

**Figure 5:**
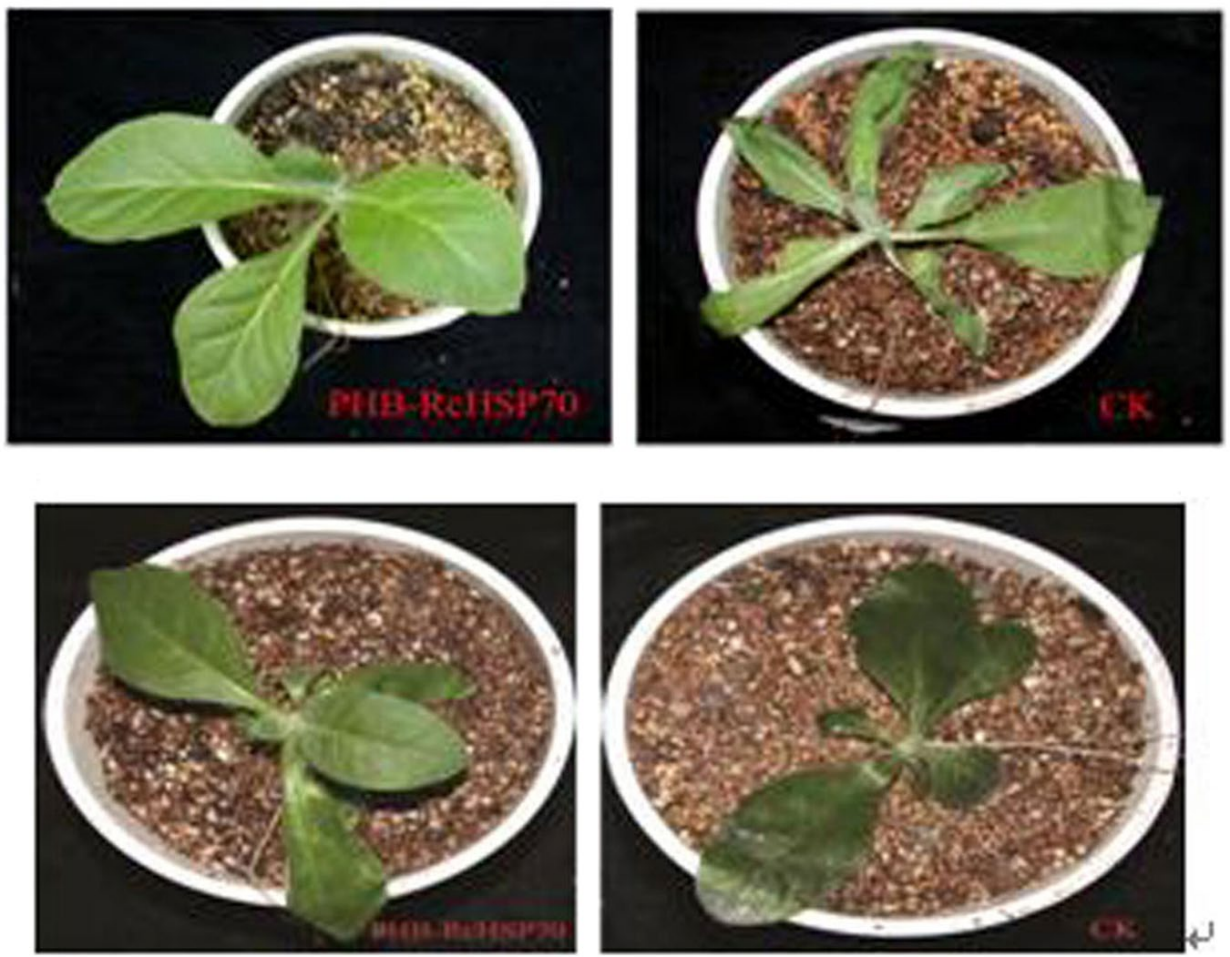
Temperature stress resistance between wild-type (CK) and transgenic tobacco (PHB-RcHSP70) under high and cold stresses (upper panel - heat shock at 46 °C/2 d; lower panel – cold stress at −5 °C/2 d).

The L1 transgenic tobacco line showed little change in morphology after heat shock treatment at 46 °C/2d, whereas the wild-type tobacco underwent significant changes (Fig. 5 upper panel). Little change occurred in the morphology of transgenic tobacco line after cold stress treatment at −5 °C/2d, whereas the wild-type tobacco wilted severely (Fig. 5 lower panel). These results indicate that the transgenic tobacco overexpresses RcHSP70, improving its resistance to high temperature and cold stress.

### Analysis of physiological indexes of heat resistance in transgenic tobacco plants

#### Analysis of changes in photosynthetic indexes

**Figure 6:**
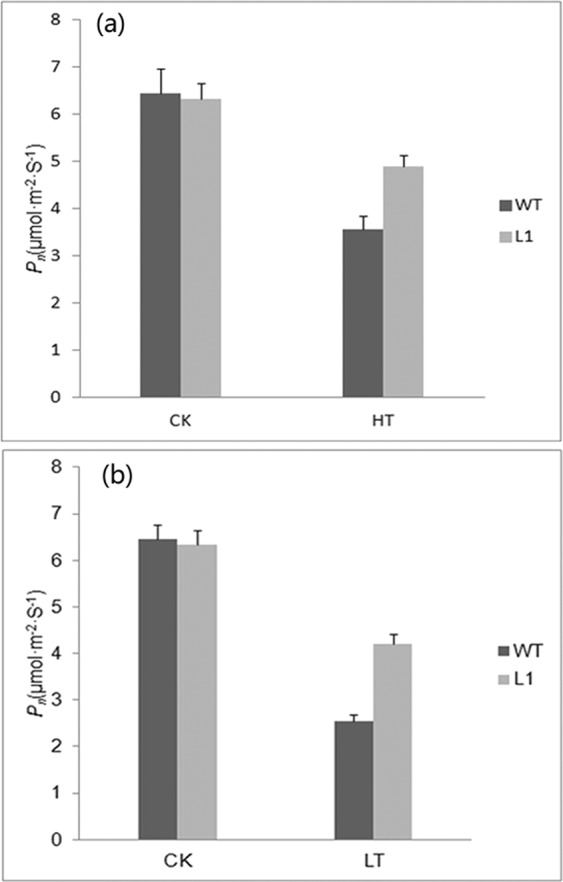
Analysis of the net photosynthetic rate (Pn) between wild-type(WT) and transgenic tobacco(L1) under high and cold stresses with nomal temperature(25 °C) as CK. (**a**) Heat shock at 46 °C/5 h (**b**) Cold stress at −5 °C/5 h.

After heat shock, the net photosynthetic rate (P_n_) of both wild-type tobacco and transgenic tobacco decreased, but the decrease rate of transgenic tobacco was significantly lower than that of wild-type tobacco (Fig. 6a), while after cold stress, they all decreased, but in the same way, the decrease rate of transgenic tobacco was significantly lower than that of wild-type tobacco (Fig. 6b).

**Figure 7:**
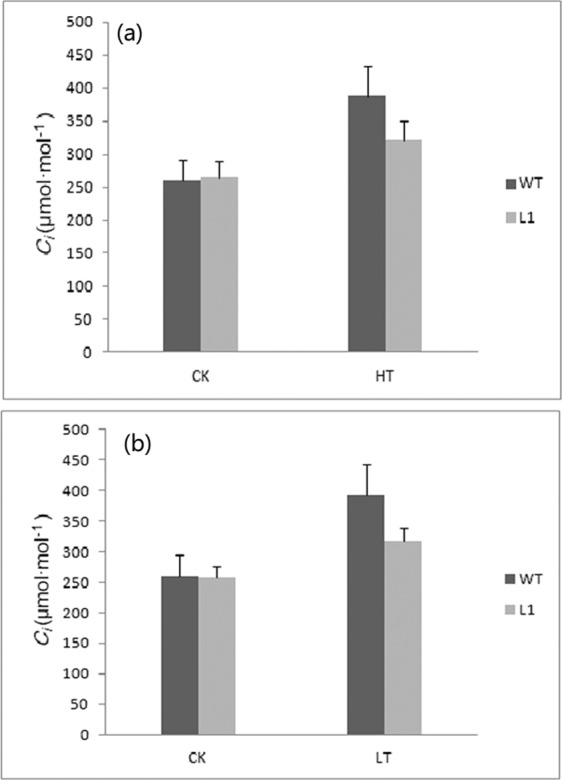
Analysis of the intercellular carbon dioxide concentration (Ci) between wild-type(WT) and transgenic tobacco(L1)under high and cold stresses with nomal temperature(25 °C) as CK (**a**). Heat shock at 46 °C/5 h (**b**) Cold stress at −5 °C/5 h.

After heat shock and cold stress, the intercellular carbon dioxide concentration (Ci) of both wild-type tobacco and transgenic tobacco increased, but the increase the rate of transgenic tobacco was significantly lower than that of wild-type tobacco(Fig. 7a,b).

**Figure 8:**
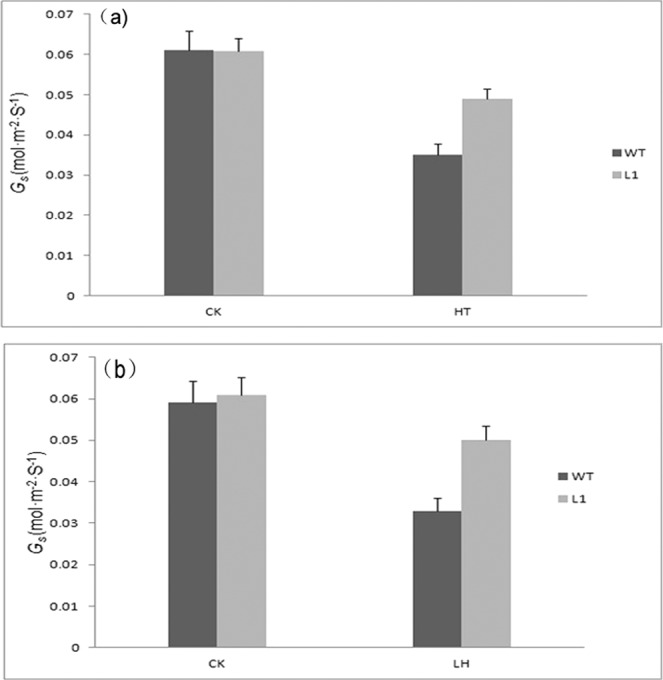
Analysis of the stomatal conductance (Gs) between wild-type(WT) and transgenic tobacco(L1) under high and cold stresses with nomal temperature(25 °C) as CK (**a**) Heat shock at 46 °C/5 h (**b**) Cold stress at −5 °C/5 h.

After heat shock and cold stress, the stomatal conductance (Gs) of both wild-type tobacco and transgenic tobacco decreased, but the decrease rate of transgenic tobacco was significantly lower than that of wild-type tobacco (Fig. 8a,b)

These results indicated that the overexpression of RcHSP70 in transgenic tobacco L1 increased its stress resistance and thus protected its photosynthetic system.

#### Change of relative electrical conductivity

**Figure 9:**
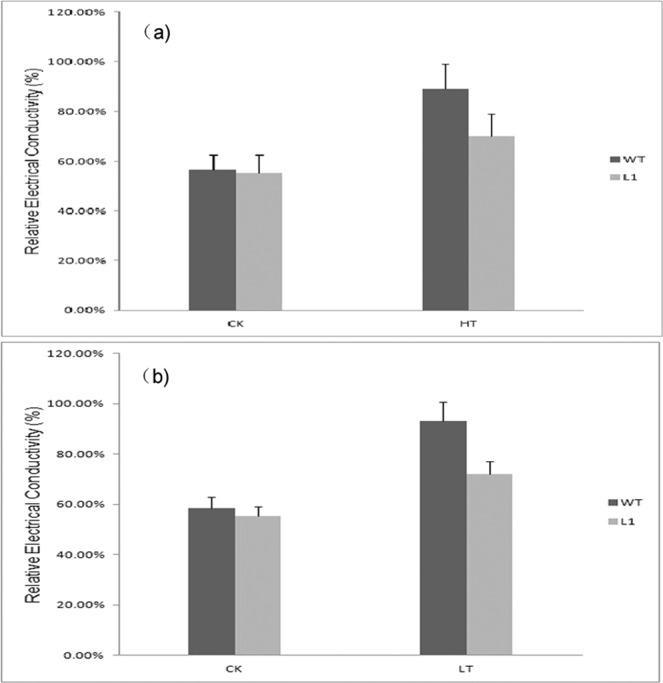
Analysis of relative electrical conductivity between wild-type(WT) and transgenic tobacco(L1) under high and cold stresses with nomal temperature(25 °C) as CK. (**a**) Heat shock at 46 °C °C5 h (**b**) Cold stress at −5 °C/5 h.

Figure 9 showed under normal control condition (CK), the relative electrical conductivity between wild tobacco and transgenic tobacco L1 had no significant difference, but following high temperature (46 °C/5 h) and lower temperature (−5/5 h) stresses, they both increase, while the increase rate of the transgenic tobacco was less than that of the wild tobacco. This result indicated that the transgenic tobacco plants had reduced cell membrane permeability and leakage of plant electrolytes under stress, thereby protecting the integrity of the cells.

#### Change of proline content

**Figure 10:**
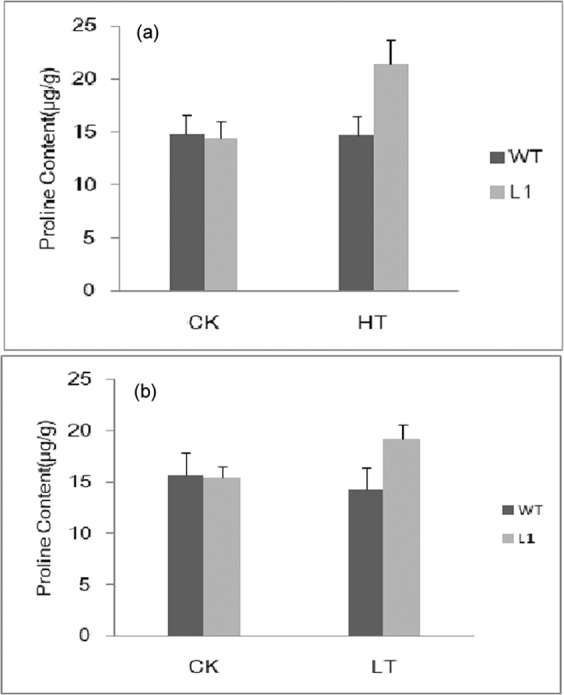
Analysis of proline content between wild-type(WT) and transgenic tobacco(L1) under high and cold stresses with nomal temperature(25 °C) as CK (**a**) Heat shock at 46 °C/5 h (**b**) Cold stress at −5 °C/5 h.

Figure 10 showed that no significant changes were observed in wild-type tobacco and transgenic tobacco under normal control condition (CK), but following high temperature (46 °C/5 h) and lower temperature (−5/5 h), the leaf proline content generally did not change in wild-type tobacco, but significantly increased in transgenic tobacco L1. These results indicated that after transformation with *RcHSP70*, the transgenic tobacco seedlings were able to increase their proline content under high temperature and lower temperature stresses.

## Discussion

Based on its expression, HSP70 can be constitutive or inducible. Inducible HSP70 is present in stressed cells; it is lowly expressed or absent in normal cells, but its level increases rapidly under heat shock or other stress conditions^10,14^. Chinese rose *RcHSP70* undergoes heat shock-induced expression; however, under high temperature stress, *RcHSP70* was highly expressed only in the heat-tolerant Chinese rose varieties—not in the non-heat-tolerant varieties. The expression of this gene is in agreement with field observations of heat tolerance in Chinese rose varieties^6–9,11^. These findings indicate that the expression of *RcHSP70* is directly related to heat tolerance in Chinese rose varieties.

HSP60 was first characterized as a molecular chaperone, and so far, HSP90, HSP70, and sHSP have been shown to function as molecular chaperones^12,17^. Studies have shown that after cells are heated, HSP70 and sHSP attach to the plasma membrane and vesicular membrane in the form of peripheral membrane proteins; these proteins can interact with membrane proteins to prevent membrane protein denaturation, stabilize the membrane system, and protect membrane microcapsules from heat^13,15^.

To verify the function of *RcHSP70*, we transformed this gene into *E. coli* BL21. The results showed that the recombinant strain improved its stress tolerance to high and low temperatures compared with the control strain. Additionally, the resistance to other abiotic stresses, such as high salt, high pH, heavy metals, and oxidation, was greater in the recombinant strain. Therefore, we believe that the improved resistance of *E. coli* to multiple stresses is directly related to *RcHSP70* expression. Because of the overexpression of RcHSP70, transgenic tobacco also improved its resistance to high temperature and cold stress. Our findings indicate that *RcHSP70* participates in the response to a variety of abiotic stresses.

When plants are under stress, their net photosynthetic rate (*P*_*n*_) decreases, stomatal conductance(*G*_*s*_) decreases, and intercellular carbon dioxide concentration(*C*_*i*_) increases. The decrease or increase degree of resistant varieties is significantly lower than that of sensitive varieties^16^.

The level of electrical conductivity is a physiological indicator commonly used to measure the extent of damage to plants under temperature stresses. High-temperature stress destroys the integrity of plant cell membranes and directly leads to leakage of cell electrolytes, thereby causing an increase in electrical conductivity^6^.

In a high-salt or drought environment, to alleviate the water imbalance caused by high-temperature stress, plants usually accumulate some organic substances in their cells, such as proline, betaine, and osmotins, that can reduce the cell water potential and ensure normal physiological function of the cells^18^. It has been reported that proline maintains the structure of proteins in cells, and also maintains the cytoplasmic pH, thereby protecting cells from damage^19^. Due to the overexpression of RcHSP70, proline content of transgenic tobacco L1 increased under heat shock and cold stress, thus enhancing the resistance.

There are no studies on Chinese rose *HSP70*. Our results should encourage the introduction of this gene into non-heat-tolerant Chinese rose varieties to improve the resistance of Chinese rose to heat and an examination of the underlying mechanisms. This work also provides theoretical support for the introduction and screening of heat-tolerant varieties of garden ornamental plants, besides Chinese rose.
